# Supplementary material for: The Impact of Harsh Stratospheric Conditions on Survival and Antibiotic Resistance Profile of Non-Spore Forming Multidrug Resistant Human Pathogenic Bacteria Causing Hospital-Associated Infections
Source: Int J Environ Res Public Health. 2023 Feb 4;20(4):2787. doi: 10.3390/ijerph20042787 (PMC9956888; doi:10.3390/ijerph20042787)
Supplement: Supplementary file 1 [file ijerph-20-02787-s001.zip › ijerph-2155811-supplementary.pdf]

Supplementary Materials

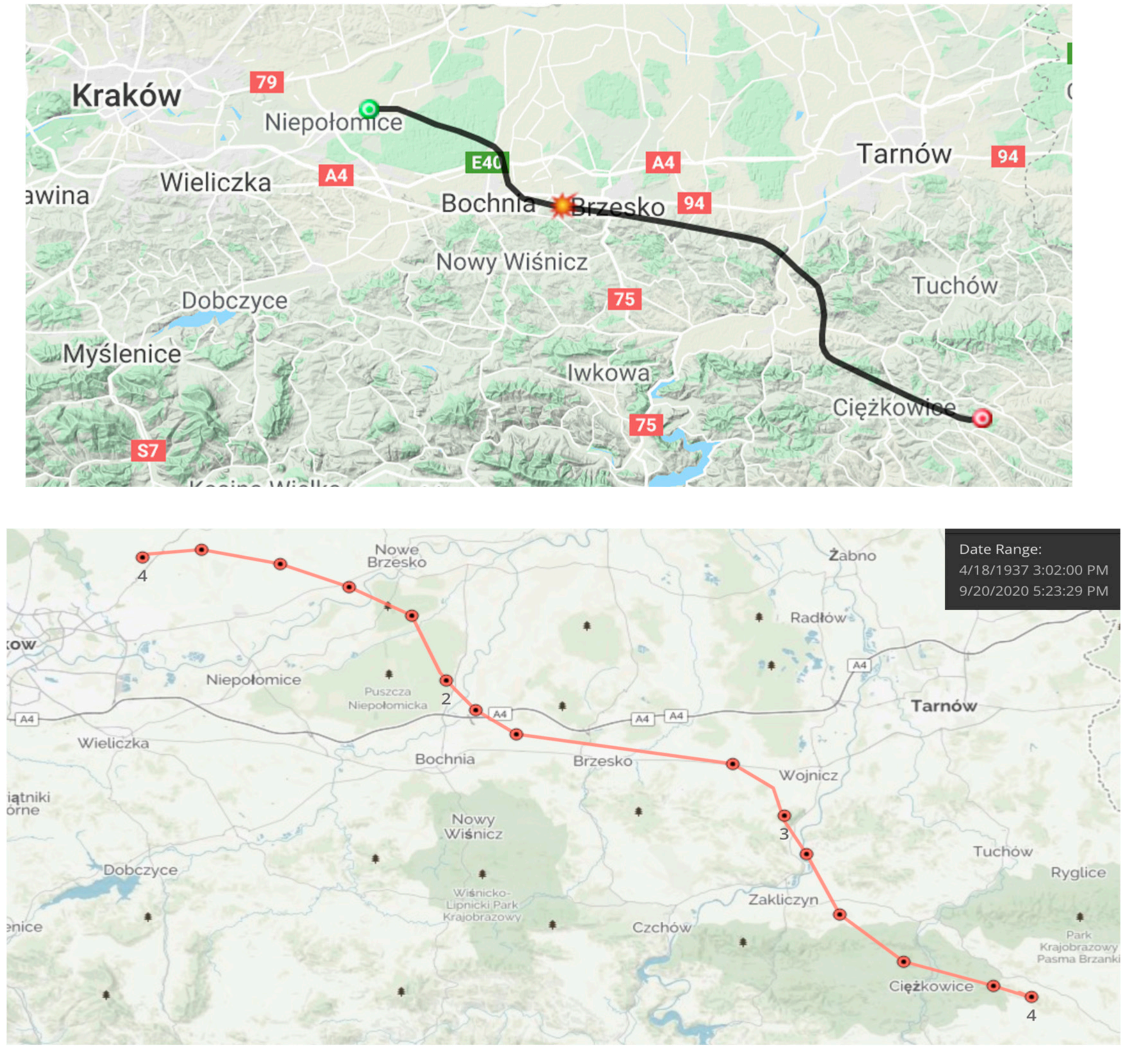

Figure S1. Maps revealing the flight prediction (top) and the flight tracking using SPOT GEN3 satellite tracking system during the STRATOS mission on 18th July 2020 (bottom).

Table S1. List of tested antibiotics used for each species of bacteria (according to EUCAST recommendations).

	E. coli	*K. pneumoniae*	*P. aeruginosa*	*E. faecium*	*S. aureus*
**Diffusion Discs**					
Amikacin 30 µg	+	+	+		+
Amoxicillin/ clavulanic acid 20/10 µg	+	+			
Ampicillin 10 µg				+	
Cefepime 30 µg	+	+	+		
Cefotaxime 30 µg	+	+			
Cefoxitin 30 µg					+
Ceftazidime 30 µg	+	+	+		
Ciprofloxacin 5 µg	+	+	+	+	+
Clindamycin 2 µg					+
Ertapenem 10 µg	+	+			
Erythromycin 15 µg					+
Gentamicin 10 µg	+	+			+
Gentamicin 30 µg				+	
Imipenem 10 µg	+	+	+	+	
Levofloxacin 5 µg	+	+	+	+	+
Lincomycin 15 µg					+
Linezolid 30 µg					+

Meropenem 10 µg	+	+	+		
Mupirocin 200 µg					+
Penicillin 30 µg					+
Piperacillin 100 µg	+	+	+		
Piperacillin/Tazobactam 100/10 µg	+	+	+		
Streptomycin 300 µg				+	
Ticarcillin 75 µg			+		
Ticarcillin/clavulanic acid 85 µg			+		
Tigecycline 15 µg	+				+
**E-tests**					
Ceftaroline range 0.002-32 µg/ml					+
Daptomycin range 0.016-256 µg/ml					+
Linezolid range 0.016-256 µg/ml				+	+
Teicoplanin range 0.016-256 µg/ml				+	+
Tigecycline range 0.016-256 µg/ml	+	+		+	+
Vancomycin range 0.016-256 µg/ml				+	+

Table S2. Details of the PCR analyses.

Gene	Primers' sequence 5' → 3'	Molecular size (bp)	Reaction protocol	Reference
kpc	F: CGTCTAGTTCTGCTGTCTTG<br>R: CTTGTCATCCTTGTTAGGCG	798	T_1: 5 min, 95°C; T_2: 30 s, 95°C; T_3: 40 s, 52°C;<br>T_4: 50 s, 72°C; T_5: 7 min, 72°C<br>T_{2-4} repeated 35 times	45
ndm-1	F: GGTTTGGCGATCTGGTTTTC<br>R: CGGAATGGCTCATCACGATC	621	T_1: 5 min, 95°C; T_2: 30 s, 95°C; T_3: 40 s, 52°C;<br>T_4: 50 s, 72°C; T_5: 7 min, 72°C<br>T_{2-4} repeated 30 times	46
vim	F: GATGGTGTTGGTCGCATA<br>R: CGAATGCGCAGCACCAG	390	T_1: 5 min, 95°C; T_2: 30 s, 95°C; T_3: 30 s, 52°C;<br>T_4: 50 s, 72°C; T_5: 7 min, 72°C<br>T_{2-4} repeated 30 times	46
*van*B	B_1: ATGGGAAGCCGATAGTC<br>B_2: GATTTCGTTCCTCGACC	635	T_1: 5 min, 95°C; T_2: 30 s, 95°C; T_3: 45 s, 54°C;<br>T_4: 1 min, 72°C; T_5: 7 min, 72°C<br>T_{2-4} repeated 30 times	47
*mec*A	F: CATTGATCGCAACGTTCAATTT<br>R: TGGTCTTTCTGCATTCCTGGA	99	T_1: 55 min, 95°C; T_2: 30 s, 95°C; T_3: 30 s, 60°C;<br>T_4: 30 s, 72°C; T_5: 10 min, 72°C<br>T_{2-4} repeated 35 times	48

Table S3. Results of the antibiotic resistance of the initial strains (before the stratospheric balloon flight).

a)

**Antibiotic**	***E. coli***		***E. coli* (resistant)**	
	**Diameter (mm)**	**EUCAST Interpretation**	**Diameter (mm)**	**EUCAST Interpretation**
AK	22	Susceptible	16	Resistant
AMC	23	Susceptible	6	Resistant
CAZ	30	Susceptible	6	Resistant
CIP	34	Susceptible	6	Resistant
CN	20	Susceptible	19	Susceptible
CTX	32	Susceptible	6	Resistant
ETP	34	Susceptible	6	Resistant
FEP	32	Susceptible	6	Resistant
IMP	30	Susceptible	8	Resistant
LEV	32	Susceptible	6	Resistant
MEM	31	Susceptible	6	Resistant
PRL	28	Susceptible	6	Resistant
TGC	25	Susceptible	24	Susceptible
TZP	28	Susceptible	6	Resistant
**E-TEST**	**MIC (mg/L)**	**EUCAST Interpretation**	**MIC (mg/L)**	**EUCAST Interpretation**
TGC	0.094	Susceptible	0,019	Susceptible

Legend: Diffusion Discs: AK – Amikacin; AMC - Amoxicillin/clavulanic acid; CAZ – Ceftazidime; CIP – Ciprofloxacin; CN – Gentamicin; CTX – Cefotaxime; ETP – Ertapenem; FEP – Cefepime; IMP – Imipenem; LEV – Levofloxacin; MEM – Meropenem; PRL – Piperacillin; TGC – Tigecycline; TZP - Piperacillin/Tazobactam; E-tests: TGC – Tigecycline

b)

Antibiotic	*K. pneumoniae*		*K. pneumoniae* **(resistant)**	
	**Diameter (mm)**	**EUCAST Interpretation**	**Diameter (mm)**	**EUCAST Interpretation**
AK	23	Susceptible	13	Resistant
AMC	17	Resistant	7	Resistant
CAZ	15	Resistant	6	Resistant
CIP	25	Susceptible	6	Resistant
CN	8	Resistant	19	Susceptible
CTX	20	Susceptible	6	Resistant
ETP	29	Susceptible	9	Resistant
FEP	25	Intermediate	6	Resistant
IMP	28	Susceptible	10	Resistant
LEV	21	Intermediate	8	Resistant
MEM	29	Susceptible	11	Resistant
PRL	10	Resistant	6	Resistant
TZP	22	Susceptible	7	Resistant
**E-TEST**	**MIC (mg/L)**	**EUCAST Interpretation**	**MIC (mg/L)**	**EUCAST Interpretation**
TGC*	3	Susceptible	1.5	Susceptible

Legend: Diffusion Discs: AK – Amikacin; AMC - Amoxicillin/ clavulanic acid; CAZ – Ceftazidime; CIP – Ciprofloxacin; CN – Gentamicin; CTX – Cefotaxime; ETP – Ertapenem; FEP – Cefepime; IMP – Imipenem; LEV – Levofloxacin; MEM – Meropenem; PRL – Piperacillin; TZP - Piperacillin/Tazobactam; E-tests: TGC – Tigecycline; * - according to EUCAST, epidemiological cut off (ECOFF) for tigecycline for *K. pneumoniae* ≥4 mg/L

c)

Antibiotic	*P. aeruginosa*		*P. aeruginosa* (resistant)	
	**Diameter (mm)**	**EUCAST Interpretation**	**Diameter (mm)**	**EUCAST Interpretation**
AK	22	Susceptible	6	Resistant
CAZ	21	Intermediate	6	Resistant
CIP	29	Intermediate	6	Resistant
FEP	23	Intermediate	6	Resistant
IMP	22	Intermediate	6	Resistant
LEV	22	Intermediate	6	Resistant
MEM	27	Susceptible	6	Resistant
PRL	29	Intermediate	6	Resistant
TIC	22	Intermediate	6	Resistant
TTC	20	Intermediate	6	Resistant
TZP	29	Intermediate	6	Resistant

Legend: Diffusion Discs: AK – Amikacin; CAZ – Ceftazidime; CIP – Ciprofloxacin; FEP – Cefepime; IMP – Imipenem; LEV – Levofloxacin; MEM – Meropenem; PRL – Piperacillin; TIC – Ticarcillin; TTC – Ticarcillin/clavulanic acid; TZP - Piperacillin/Tazobactam

d)

Antibiotic	*E. faecalis*		*E. faecalis* **(resistant)**	
	**Diameter (mm)**	**EUCAST Interpretation**	**Diameter (mm)**	**EUCAST Interpretation**
AMP	24	Susceptible	29	Susceptible
CIP	21	Susceptible	24	Susceptible
CN 30	21	HLGR (-)	6	HLGR (+)
IMP	27	Intermediate	30	Intermediate
LEV	20	Susceptible	22	Susceptible
S 300	19	HLSR (-)	17	HLSR (-)
**E-TEST**	**MIC (mg/L)**	**EUCAST Interpretation**	**MIC (mg/L)**	**EUCAST Interpretation**
LZ	1.5	Susceptible	1.5	Susceptible
TGC	0.094	Susceptible	0.094	Susceptible
TP	0.19	Susceptible	0.75	Susceptible
VA	4.0	Susceptible	48	Resistant

Legend: Diffusion Discs: AMP – Ampicillin; CIP – Ciprofloxacin; CN – Gentamicin; IMP – Imipenem; LEV – Levofloxacin; S – Streptomycin; E-tests: LZ – Linezolid; TGC – Tigecycline; TP – Teicoplanin; VA – Vancomycin

e)

Antibiotic	*S. aureus*		*S. aureus* (MRSA/VSSA)		*S. aureus* (MRSA/VISA)	
	**Diameter (mm)**	**EUCAST Interpretation**	**Diameter (mm)**	**EUCAST Interpretation**	**Diameter (mm)**	**EUCAST Interpretation**
AK	21	Susceptible	14	Resistant	14	Resistant
CIP	29	Intermediate	24	Intermediate	6	Resistant
CN	22	Susceptible	24	Susceptible	6	Resistant
DA	32	Susceptible	28	Susceptible	6	Resistant
E	20	Susceptible	6	Resistant	6	Resistant
FOX	30	Susceptible	9	Resistant	6	Resistant
LEV	30	Intermediate	26	Intermediate	8	Resistant
LZD	32	Susceptible	32	Susceptible	34	Susceptible
MLS-B	-	Sensitive MLS-B	+	Inducible MLS-B	-	Constitutive MLS-B
MUP	42	Susceptible	6	Resistant	45	Susceptible
MY	32	Susceptible	32	Susceptible	6	Resistant
P	34	Susceptible	8	Resistant	6	Resistant
TGC	20	Susceptible	25	Susceptible	30	Susceptible
**E-TEST**	**MIC (mg/L)**	**EUCAST Interpretation**	**MIC (mg/L)**	**EUCAST Interpretation**	**MIC (mg/L)**	**EUCAST Interpretation**
CPT	0.19	Susceptible	0.75	Susceptible	1.50	Intermediate
DPC	0.38	Susceptible	0.25	Susceptible	1.0	Susceptible
LZ	1.5	Susceptible	1.5	Susceptible	1.0	Susceptible
TGC	0.094	Susceptible	0.125	Susceptible	0.125	Susceptible
TP	1.5	Susceptible	1	Susceptible	8.0	Resistant
VA	2.0	Susceptible	1	Susceptible	4.0	Resistant

Legend: MRSA/VSSA – methicillin resistant *Staphylococcus aureus*/vancomycin sensitive *S. aureus*; MRSA/VISA - methicillin resistant *Staphylococcus aureus*/vancomycin intermediate *S. aureus*; Diffusion Discs: AK – Amikacin; CIP – Ciprofloxacin; CN – Gentamicin; DA – Clindamycin; E – Erythromycin; FOX – Cefoxitin; LEV – Levofloxacin; LZD – Linezolid; MLS-B – macrolide, lincosamide, streptogramine B;

MUP – Mupirocin; MY – Lincomycin; P – Penicillin; TGC – Tigecycline; E-tests: CPT – Ceftaroline; DPC – Daptomycin; LZ – Linezolid; TGC – Tigecycline; TP – Teicoplanin; VA – Vancomycin

Table S4. Diameter/MIC after the flight. Shifts indicating susceptibility changes relative to initial results (see Table S3).

a)

E. coli							
	**Ground Control**			**Experimental Group**	**Flight Control**		
**Antibiotic**	**Diameter (mm)**	**Change**	**Susceptibility**		**Diameter (mm)**	**Change**	**Susceptibility**
CIP	29	↓	↓	No Growth	38	↑	↑
IMP	16	↓	↓		34	↑	↑
PRL	21	↓	↓		32	↑	↑
CN	17	↓	↓		24	↑	↑
AMC	20	↓	↓		23	n/c	n/c
ETP	26	↓	↓		28	↓	↓
AK	17	↓	↓		24	↑	↑
MEM	25	↓	↓		39	↑	↑
CTX	27	↓	↓		38	↑	↑
LEV	28	↓	↓		34	↑	↑
TZP	22	↓	↓		32	↑	↑
CAZ	24	↓	↓		40	↑	↑
FEP	27	↓	↓		38	↑	↑
TGC	21	↓	↓		30	↑	↑
**E-TEST**	**MIC (mg/L)**	**Change**	**Susceptibility**		**MIC (mg/L)**	**Change**	**Susceptibility**
TGC	0,125	↑	↓	No Growth	0,19	↑	↓

b)

E. coli (CPE)									
	Ground Control			Experimental Group			Flight Control		
**Antibiotic**	**Diameter (mm)**	**Change**	**Susceptibility**	**Diameter (mm)**	**Change**	**Susceptibility**	**Diameter (mm)**	**Change**	**Susceptibility**
CIP	6	n/c	n/c	6	n/c	n/c	6	n/c	n/c
IMP	6	↓	↓	6	↓	↓	6	↓	↓
PRL	6	n/c	n/c	6	n/c	n/c	6	n/c	n/c
CN	17	↓	↓	20	↓	↓	20	↑	↑
AMC	6	n/c	n/c	6	n/c	n/c	6	n/c	n/c
ETP	6	n/c	n/c	6	n/c	n/c	6	n/c	n/c
AK	15	↓	↓	15	↓	↓	16	n/c	n/c
MEM	6	n/c	n/c	6	n/c	n/c	6	n/c	n/c
CTX	6	n/c	n/c	12	↑	↑	8	↑	↑
LEV	6	n/c	n/c	6	n/c	n/c	6	n/c	n/c
TZP	6	n/c	n/c	12	↑	↑	6	n/c	n/c
CAZ	6	n/c	n/c	14	↑	↑	11	↑	↑
FEP	6	n/c	n/c	10	↑	↑	6	n/c	n/c
TGC	18	↓	↓	24	n/c	n/c	22	↓	↓
**E-TEST**	**MIC (mg/L)**	**Change**	**Susceptibility**	**MIC (mg/L)**	**Change**	**Susceptibility**	**MIC (mg/L)**	**Change**	**Susceptibility**
TGC	0,38	↑	↓	0,25	↑	↓	0,38	↑	↓

c)

					K. pneumoniae				
	**Ground Control**			**Experimental Group***			**Flight Control**		
**Antibiotic**	**Diameter (mm)**	**Change**	**Susceptibility**	**Diameter (mm)**	**Change**	**Susceptibility**	**Diameter (mm)**	**Change**	**Susceptibility**
CIP	20	↑	↑	42	↑	↑	21	↓	↓
IPM	23	↓	↓	44	↑	↑	28	n/c	n/c
PRL	6	↓	↓	22	↑	↑	10	n/c	n/c
CN	12	↑	↑	22	↑	↑	13	↑	↑
AMC	17	n/c	n/c	32	↑	↑	17	n/c	n/c
ETP	25	↓	↓	34	↑	↑	24	↓	↓
AK	20	↓	↓	38	↑	↑	18	↓	↓
MEM	23	↓	↓	32	↑	↑	29	n/c	n/c
CTX	17	↓	↓	38	↑	↑	17	↓	↓
LEV	18	↓	↓	30	↑	↑	19	↓	↓
TZP	19	↓	↓	24	↑	↑	20	↓	↓
CAZ	12	↓	↓	18	↑	↑	13	↓	↓
FEP	21	↓	↓	34	↑	↑	22	↓	↓
**E-TEST**	**MIC (mg/L)**	**Change**	**Susceptibility**				**MIC (mg/L)**	**Change**	**Susceptibility**
TGC	4	↑	↓		No Growth		6	↑	↓

d)

K. pneumoniae (CPE)									
	**Ground Control**			**Experimental Group***			**Flight Control**		
**Antibiotic**	**Diameter (mm)**	**Change**	**Susceptibility**	**Diameter (mm)**	**Change**	**Susceptibility**	**Diameter (mm)**	**Change**	**Susceptibility**
CIP	6	n/c	n/c	18	↑	↑	6	n/c	n/c
IPM	6	↓	↓	28	↑	↑	6	↓	↓
PRL	6	n/c	n/c	- **	-	-	6	n/c	n/c
CN	17	↓	↓	- **	-	-	17	↓	↓
AMC	6	↓	↓	30	↑	↑	6	↓	↓
ETP	6	↓	↓	- **	-	-	6	↓	↓
AK	11	↓	↓	- **	-	-	12	↓	↓
MEM	6	n/c	n/c	26	↑	↑	6	n/c	n/c
CTX	6	n/c	n/c	16	↑	↑	6	n/c	n/c
LEV	8	↑	↑	42	↑	↑	6	n/c	n/c
TZP	6	n/c	n/c	42	↑	↑	6	n/c	n/c
CAZ	6	n/c	n/c	30	↑	↑	6	n/c	n/c
FEP	6	n/c	n/c	24	↑	↑	6	n/c	n/c
**E-TEST**	**MIC (mg/L)**	**Change**	**Susceptibility**				**MIC (mg/L)**	**Change**	**Susceptibility**
TGC	1,5	n/c	n/c		No Growth		1,5	n/c	n/c

e)

				P. aeruginosa			
	Ground Control			Experimental Group	Flight Control		
**Antibiotic**	**Diameter (mm)**	**Change**	**Susceptibility**		**Diameter (mm)**	**Change**	**Susceptibility**
AK	24	↑	↑	No Growth	28	↑	↑
CIP	35	↑	↑		34	↑	↑
LEV	26	↑	↑		30	↑	↑
CAZ	29	↑	↑		24	↑	↑
FEP	32	↑	↑		36	↑	↑
IPM	18	↓	↓		32	↑	↑
MEM	17	↓	↓		38	↑	↑
TTC	19	↓	↓		30	↑	↑
TZP	32	↑	↑		36	↑	↑
PRL	26	↓	↓		36	↑	↑
TIC	26	↑	↑		26	↑	↑

f)

P. aeruginosa (MDR)							
	**Ground Control**			**Experimental Group**	**Flight Control**		
**Antibiotic**	**Diameter (mm)**	**Change**	**Susceptibility**		**Diameter (mm)**	**Change**	**Susceptibility**
AK	6	n/c	n/c	No Growth	6	n/c	n/c
CIP	6	n/c	n/c		6	n/c	n/c
LEV	6	n/c	n/c		6	n/c	n/c
CAZ	10	↑	↑		10	↑	↑
FEP	8	↑	↑		9	↑	↑
IPM	6	n/c	n/c		6	n/c	n/c
MEM	6	n/c	n/c		6	n/c	n/c
TTC	6	n/c	n/c		6	n/c	n/c
TZP	14	↑	↑		16	↑	↑
PRL	14	↑	↑		34	↑	↑
TIC	6	n/c	n/c		6	n/c	n/c

g)

E. faecalis									
	**Ground Control**			**Experimental Group**			**Flight Control**		
**Antibiotic**	**Diameter (mm)**	**Change**	**Susceptibility**	**Diameter (mm)**	**Change**	**Susceptibility**	**Diameter (mm)**	**Change**	**Susceptibility**
CIP	21	n/c	n/c	28	↑	↑	20	↓	↓
IPM	28	↑	↑	34	↑	↑	27	n/c	n/c
AMP	22	↓	↓	32	↑	↑	27	↑	↑
GN	20	↓	↓	24	↑	↑	18	↓	↓
S	16	↓	↓	22	↑	↑	16	↓	↓
LEV	21	↑	↑	24	↑	↑	19	↓	↓
**E-TEST**	**MIC (mg/L)**	**Change**	**Susceptibility**				**MIC (mg/L)**	**Change**	**Susceptibility**
TP	0,19	n/c	n/c	No Growth			0,38	↑	↓
VA	4	n/c	n/c				4	n/c	n/c
TGC	0,125	↑	↓				0,094	n/c	n/c
LZ	1,5	n/c	n/c				3	↑	↓

h)

	E. faecalis (VRE)								
	**Ground Control**			**Experimental Group**			**Flight Control**		
**Antibiotic**	**Diameter (mm)**	**Change**	**Susceptibility**	**Diameter (mm)**	**Change**	**Susceptibility**	**Diameter (mm)**	**Change**	**Susceptibility**
CIP	24	n/c	n/c	28	↑	↑	23	↓	↓
IPM	28	↓	↓	30	n/c	n/c	26	↓	↓
AMP	28	↓	↓	26	↓	↓	28	↓	↓
GN	6	n/c	n/c	6	n/c	n/c	6	n/c	n/c
S	17	n/c	n/c	26	↑	↑	12	↓	↓
LEV	24	↑	↑	28	↑	↑	22	n/c	n/c
**E-TEST**	**MIC (mg/L)**	**Change**	**Susceptibility**				**MIC (mg/L)**	**Change**	**Susceptibility**
TP	0,5	↓	↑				0,75	n/c	n/c
VA	48	n/c	n/c		No Growth		48	n/c	n/c
TGC	0,094	n/c	n/c				0,125	↑	↓
LZ	1,5	n/c	n/c				2	↑	↓

i)

S. aureus							
	**Ground Control**			**Experimental Group**	**Flight Control**		
**Antibiotic**	**Diameter (mm)**	**Change**	**Susceptibility**		**Diameter (mm)**	**Change**	**Susceptibility**
FOX	32	↑	↑	No Growth	32	↑	↑
MUP	40	↓	↓		40	↓	↓
LZD	30	↓	↓		32	n/c	n/c
E	32	↑	↑		32	↑	↑
DA	30	↓	↓		29	↓	↓
MY	30	↓	↓		30	↓	↓
P	36	↑	↑		38	↑	↑
LEV	28	↓	↓		32	↑	↑
CIP	26	↓	↓		28	↓	↓
CN	22	n/c	n/c		22	n/c	n/c
AK	19	↓	↓		20	↓	↓
TGC	27	↓	↓		28	↓	↓
**E-TEST**	**MIC (mg/L)**	**Change**	**Susceptibility**		**MIC (mg/L)**	**Change**	**Susceptibility**
VA	3,0	↑	↓	No Growth	2,0	n/c	n/c
TP	1,5	n/c	n/c		1,5	n/c	n/c
CPT	0,047	↓	↑		0,047	↓	↑
TGC	0,064	↓	↑		0,094	n/c	n/c
LZ	1,5	n/c	n/c		1,0	↓	↑
DPC	0,038	↓	↑		0,25	↓	↑

j)

S. aureus (MRSA/VSSA)									
	**Ground Control**			**Experimental Group**			**Flight Control**		
**Antibiotic**	**Diameter (mm)**	**Change**	**Susceptibility**	**Diameter (mm)**	**Change**	**Susceptibility**	**Diameter (mm)**	**Change**	**Susceptibility**
FOX	8	↓	↓	- **	-	-	8	↓	↓
MUP	6	n/c	n/c	- **	-	-	6	n/c	n/c
LZD	26	↓	↓	44	↑	↑	27	↓	↓
E	6	n/c	n/c	- **	-	-	6	n/c	n/c
DA	6	↓	↓	- **	-	-	6	↓	↓
MY	6	↓	↓	- **	-	-	6	↓	↓
P	8	n/c	n/c	20	↑	↑	6	↓	↓
LEV	20	↓	↓	- **	-	-	28	↑	↑
CIP	21	↓	↓	35	↑	↑	24	n/c	n/c
GN	22	↓	↓	30	↑	↑	23	↓	↓
AK	14	n/c	n/c	16	↑	↑	13	↓	↓
TGC	22	↓	↓	32	↑	↑	24	↓	↓
**E-TEST**	**MIC (mg/L)**	**Change**	**Susceptibility**	**MIC (mg/L)**	**Change**	**Susceptibility**	**MIC (mg/L)**	**Change**	**Susceptibility**
VA	2,0	↑	↓	1,5	↑	↓	1,5	↑	↓
TP	1,0	n/c	n/c	0,25	↓	↑	1,5	↑	↓
CPT	0,75	n/c	n/c	0,25	↓	↑	0,75	n/c	n/c
TGC	0,125	n/c	n/c	0,094	↓	↑	0,19	↑	↓
LZ	1,5	n/c	n/c	0,064	↓	↑	1,5	n/c	n/c
DPC	0,38	↑	↓	0,125	↓	↑	0,25	n/c	n/c

k)

	S. aureus (MRSA/VISA)						
	Ground Control			Experimental Group	Flight Control		
**Antibiotic**	**Diameter (mm)**	**Change**	**Susceptibility**		**Diameter (mm)**	**Change**	**Susceptibility**
FOX	6	n/c	n/c	No Growth	6	n/c	n/c
MUP	42	↓	↓		42	↓	↓
LZD	36	↑	↑		34	n/c	n/c
E	6	n/c	n/c		6	n/c	n/c
DA	6	n/c	n/c		6	n/c	n/c
MY	6	n/c	n/c		6	n/c	n/c
P	6	n/c	n/c		6	n/c	n/c
LEV	11	↑	↑		9	↓	↓
CIP	6	n/c	n/c		6	n/c	n/c
GN	6	n/c	n/c		6	n/c	n/c
AK	13	↓	↓		11	↓	↓
TGC	28	↓	↓		27	↓	↓
**E-TEST**	**MIC (mg/L)**	**Change**	**Susceptibility**		**MIC (mg/L)**	**Change**	**Susceptibility**
VA	6,0	↑	↓	No Growth	4,0	n/c	n/c
TP	12,0	↑	↓		12,0	↑	↓
CPT	2,0	↑	↓		2,0	↑	↓
TGC	0,125	n/c	n/c		0,25	↑	↓
LZ	1,0	n/c	n/c		1,0	n/c	n/c
DPC	1,0	n/c	n/c		1,0	n/c	n/c

Legend: Abbreviations and special indications for the tables:
AK – Amikacin; AMC - Amoxicillin/clavulanic acid; CAZ – Ceftazidime; CIP – Ciprofloxacin; CN – Gentamicin; CTX – Cefotaxime; ETP – Ertapenem; FEP – Cefepime; IMP – Imipenem; LEV – Levofloxacin; MEM – Meropenem; PRL – Piperacillin; TGC – Tigecycline; TZP - Piperacillin/Tazobactam; TGC – Tigecycline; TIC – Ticarcillin; TTC – Ticarcillin/clavulanic acid; AMP – Ampicillin; S – Streptomycin: LZ – Linezolid; TP – Teicoplanin; VA – Vancomycin; DA – Clindamycin; E – Erythromycin; FOX – Cefoxitin; LZD – Linezolid; MLS-B – macrolide, lincosamide, streptogramine B; MUP – Mupirocin; MY – Lincomycin; P – Penicillin; CPT – Ceftaroline; DPC – Daptomycin; n/c – not changed;
*- Due to small growth rate radiuses from the nearest colony were calculated
**- Reading impossible due to technical issues

Table S5. Changes in clinical interpretation after the stratospheric balloon flight (according to EUCAST recommendations).

Bacterial Strain	Antibiotic (Diffusion Disc)	Before Flight		After Flight		
		Diameter (mm)	Interpretation	Diameter (mm)	Interpretation	Group
E. coli	Imipenem	30	Sensitive	16	Resistant	Ground Control
	Amikacin	22	Sensitive	17	Resistant	Ground Control
K. pneumoniae	Ciprofloxacin	25	Sensitive	20; 21	Resistant	Ground Control; Flight Control
	Piperacillin	10*	Resistant	22	Sensitive	Experimental Group
	Gentamicin	8*	Resistant	22	Sensitive	Experimental Group
	Amikacin	17	Resistant	32	Sensitive	Experimental Group
	Ertapenem	29	Sensitive	24	Resistant	Flight Control
	Cefotaxime	20	Sensitive	17; 17	Intermediate	Ground Control; Experimental Group
	Levofloxacin	21	Intermediate	18; 30	Resistant; Sensitive	Ground Control; Experimental Group
	Piperacillin/Tazobactam	22	Sensitive	19	Intermediate	Ground Control
	Cefepime	25	Intermediate	21; 34	Resistant; Sensitive	Ground Control; Experimental Group
	Imipenem	10*	Resistant	28	Sensitive	Experimental Group

K. pneumoniae (resistant)	Amikacin	7	Resistant	30	Sensitive	Experimental Group
	Meropenem	11**	Resistant	26	Sensitive	Experimental Group
	Levofloxacin	8**	Resistant	42	Sensitive	Experimental Group
	Piperacillin/Tazobactam	7**	Resistant	42	Sensitive	Experimental Group
	Ceftazidime	6	Resistant	30	Sensitive	Experimental Group
	Cefepime	6	Resistant	24	Intermediate	Experimental Group
P. aeruginosa	Imipenem	22	Intermediate	18*	Resistant	Ground Control
	Meropenem	27	Sensitive	17	Resistant	Ground Control
P. aeruginosa (resistant)	Piperacillin	10**	Resistant	34	Intermediate	Flight Control
E. faecalis (resistant)	Streptomycin	17	S	12	Resistant	Flight Control

**Bacterial Strain**	**Antibiotic (E-TEST)**	**Before Flight**		**After Flight**		
		MIC Breakpoint (mg/L)	Interpretation	MIC Breakpoint (mg/L)	Interpretation	Group
S. aureus	Vancomycin	2.0	Sensitive	3.0	Resistant	Ground Control
S. aureus (MRSA/VISA)	Ceftaroline	1.5	Intermediate	2.0; 2.0	Resistant	Control Group; Flight Group

Legend: * Radius measured from the closest colony; ** Diameter of the halo around diffusion disc. Colonies within that halo were observed
